# Exploring unregulated substance use health data in Ontario, Canada: Identifying gaps, addressing challenges, and uncovering opportunities

**DOI:** 10.3389/fpubh.2025.1477539

**Published:** 2025-08-27

**Authors:** Farihah Ali, Justine Law, Cayley Russell, Sameer Imtiaz, Ahmed Bayoumi, Dan Werb, Jürgen Rehm

**Affiliations:** ^1^Centre for Addiction and Mental Health (CAMH), Institute for Mental Health Policy Research, Toronto, ON, Canada; ^2^Ontario Node, Canadian Research Initiative in Substance Matters (CRISM), Toronto, ON, Canada; ^3^Dalla Lana School of Public Health, University of Toronto, Toronto, ON, Canada; ^4^Centre on Drug Policy Evaluation, MAP Centre for Urban Health Solutions, St. Michael’s Hospital, Toronto, ON, Canada; ^5^ICES, Toronto, ON, Canada; ^6^Department of Medicine, University of Toronto, Toronto, ON, Canada; ^7^Institute of Health Policy, Management & Evaluation, University of Toronto, Toronto, ON, Canada; ^8^Division of General Internal Medicine, St. Michael’s Hospital, Toronto, ON, Canada; ^9^Institute of Medical Sciences (IMS), University of Toronto, Toronto, ON, Canada; ^10^Division of Infectious Diseases & Global Public Health, Department of Medicine, University of California San Diego, San Diego, CA, United States; ^11^Department of Psychiatry, Faculty of Medicine, University of Toronto, Toronto, ON, Canada; ^12^Campbell Family Mental Health Research Institute, CAMH, Toronto, ON, Canada; ^13^Program on Substance Abuse & WHO European Region Collaborating Centre, Public Health Agency of Catalonia, Barcelona, Spain; ^14^Center for Interdisciplinary Addiction Research, Department of Psychiatry and Psychotherapy, University Medical Center Hamburg-Eppendorf, Hamburg, Germany; ^15^Program on Substance Abuse & WHO European Region Collaborating Centre, Public Health Agency of Catalonia, Barcelona, Spain; ^16^Center for Interdisciplinary Addiction Research,Department of Psychiatry and Psychotherapy, University Medical Center Hamburg-Eppendorf, Hamburg, Germany

**Keywords:** Canada, health data management, health policy, healthcare systems, Ontario, overdose, unregulated substance use

## Abstract

Canada’s overdose epidemic underscores the urgent need for high-quality, comprehensive, and timely national health data to inform evidence-based policies, population health management, and targeted intervention strategies. Using the province of Ontario, Canada, as a case study, this paper examines the current landscape of unregulated substance use health data, including both administrative and non-administrative health data sources. Health data on unregulated substance use in Ontario are fragmented, inconsistently collected, and poorly shared across organizations and jurisdictions. This creates significant barriers for researchers and decision makers in accessing timely and reliable information. Moreover, significant gaps persist in key areas, including prevalence estimates, treatment uptake, drug use profiles, marginalized populations, and disaggregated socio-demographic data. These deficiencies reflect and compound limitations at the national level, and hinder comprehensive analyses and informed decision-making, as well as progress toward coordinated national surveillance. To address these challenges, we propose several key recommendations: (1) standardize and integrate data to enhance consistency and interoperability among data sources; (2) improve data availability and accuracy to strengthen reporting mechanisms, increase transparency, and enable real-time monitoring of substance use trends, and (3) reduce barriers to data collection, analysis, and dissemination through enhanced collaboration and innovation. These strategies will improve provincial response efforts and contribute to building a national surveillance system that supports evidence-based decision-making to more effectively address the overdose crisis.

## 1 Introduction

The drug overdose crisis has resulted in over 42,000 deaths across Canada since January 2016, with the province of Ontario accounting for over a third (36%) of these fatalities (1). The province has experienced a surge in overdose events, hospitalizations, and other harms associated with the use of unregulated drugs, emphasizing the urgent need for evidence-informed strategies that address both the clinical and social determinants of substance use (2). Effective policymaking in this area depends on comprehensive health data, capturing patterns of use, service access, diagnoses, and substance use-related harms. These data support targeted prevention, harm reduction, and treatment strategies tailored to specific populations, while enabling the evaluation of interventions over time. Together, such data offer a comprehensive understanding of the scope and consequences of unregulated substance use and its multifaceted impacts on individuals and society (2, 3).

Ontario is uniquely positioned in Canada with respect to health data capacity. It hosts the country’s largest collection of administrative health data, supplemented by survey data, strong data governance frameworks, and mature infrastructure for population health research (4). The province has also invested significantly in health data systems, most notably in establishing ICES, Canada’s largest provincial data center, which operates as both a research institute and data repository. ICES supports secure, deidentified access to individual-level health data that can be used toward population health initiatives (4–8).

Comparative analyses have found Ontario to have fewer barriers to health data access than other provinces such as Nova Scotia and British Columbia, largely due to ICES’ integrated data holdings and well-developed infrastructure (6). Ontario also maintains several specialized databases, including the Ontario Mental Health Reporting System (OMHRS), which offers detailed information on hospital admissions, patients’ mental and physical health, social supports, and service use. OMHRS includes advanced algorithms to identify key mental health and addiction indicators, thereby improving data precision (5, 8). These strengths reflect the Ontario government’s sustained commitment to data-driven health system innovation, evidenced by ongoing investments and legislative support for ICES to expand operational capacity and foster a robust research environment (6, 8).

Despite efforts to improve data collection and utilization, challenges persist in the collection, access, and dissemination of these data (3). Key barriers include data silos, limited data on marginalized populations (e.g., unhoused individuals, those in correctional institutes, or private treatment settings), inconsistent data sharing across jurisdictions, and gaps in real-time surveillance (3, 9, 10). However, these issues are not unique to Ontario; they reflect broader systemic challenges across Canada. Existing research has pointed to deficiencies in national-level surveillance systems, including the absence of timely data on emerging drug trends, demographic gaps (e.g., age, gender, ethnicity), and underrepresentation of equity-deserving communities. (3, 11–15). While previous research has described broad challenges in Canadian substance use surveillance, such as a lack of standardization and data-sharing frameworks, these studies have primarily relied on qualitative methodologies, such as key informant interviews, to describe these gaps (10, 16). Furthermore, although frameworks and strategies for data collection and surveillance measures have been explored (3, 5, 8, 15, 17–21), few studies have comprehensively mapped the various provincial data sources on unregulated substance use, or examined the structural, technical, and operational barriers affecting provincial and national surveillance capacity.

At present, Canada lacks a fully operational, coordinated national drug surveillance system that provides accessible, up-to-date, and high-quality data across jurisdictions. Advocates and policymakers have called for standardized infrastructure to support consistent collection, integration and dissemination of substance use data (2, 11). Such a harmonized national system could, track population-level health outcomes, and strengthen the capacity to assess the impact of interventions and policy changes (22). Additionally, it could serve as an early warning system to detect signals of potential future drug epidemics and respond to emerging drug trends (10, 21, 23). In response, several federal and provincial initiatives are underway (9). Health Canada is examining the feasibility of a Canadian Drugs Observatory, while the Office of Drug Research and Surveillance is collaborating with federal and provincial/territorial partners to advance integrated data systems (9).

Against this backdrop, this study maps Ontario’s unregulated substance use health data landscape, identifying available national and provincial-level health data sources, key gaps, and system-level challenges. While focused on Ontario, the findings are relevant to other Canadian jurisdictions and international settings facing similar data infrastructure limitations (6). By analyzing Ontario’s strengths and limitations, we offer recommendations to enhance data collection and dissemination, strengthen surveillance capacity, and inform the development of a national data strategy. These insights are critical to designing equitable, coordinated, and data-driven responses to Canada’s ongoing overdose crisis.

## 2 Methods

To describe the current landscape of unregulated substance use health data in Ontario, we conducted a semi-structured gray literature search between January and April 2024 using Google and key sources such as Statistics Canada, the Canadian Institute for Health Information (CIHI), ICES, and the Center for Addiction and Mental Health (CAMH). This search aimed to identify source of substance use-related health data, as well as relevant policy documents, frameworks, and reports, to better understand the broader context shaping data collection and dissemination of substance use-related health data (24, 25). We included any Ontario health data on unregulated substance use including prevalence, behaviors, harms, and treatment/service utilization. Additionally, we identified sources through backwards citation-chaining of academic articles or reports, and established tools like Public Health Ontario (PHO) Interactive Opioid Tool, the Public Health Agency of Canada’s Opioid-and Stimulant-Related Harms Interactive Tool, and the Ontario Drug Policy Research Network (ODPRN) Opioid Indicator Tool (1, 26, 27). Search terms included relevant keywords such as: ‘Substance Use’ or ‘Illicit Drug Use’ or ‘Unregulated Drug Use’, ‘Opioid’, ‘Overdose’ ‘Prevalence’, ‘Harms’, or ‘Behaviors’, ‘Data,’ ‘Databases’ or ‘Datasets’, specifying Ontario as the geographical focus. Only English-language sources were included, and no date limitations were applied. Non-health data (such as incarcerations or arrests related to unregulated substance use) were excluded. We note that data availability, collection, and access are evolving and subject to change.

## 3 Results—current unregulated substance use health data landscape in Ontario

In Ontario, health data on unregulated substance use primarily fall into two main categories: (1) administrative health data, capturing substance use-related harms and treatment outcomes (e.g., hospital admissions, emergency department (ED) visits, and prescriptions) (24), and (2) non-administrative health data from population-based surveys assessing unregulated substance use prevalence and behaviors among the general population or specific sub-populations (28).

### 3.1 Administrative unregulated substance use health data in Ontario

Administrative health data are comprised of demographic, administrative, and clinical outputs routinely collected by governments and other public (e.g., hospitals) and private (e.g., treatment) institutions (24). These outputs serve various functions within the healthcare system to facilitate the operation of health services, from managing patient flow to allocating funding, and are frequently utilized for research purposes (24).

In Ontario, administrative health data related to unregulated substance use are gathered during every interaction individuals have with the publicly-funded healthcare system, such as during ED visits, hospital admissions, doctor or clinic visits, and pharmacy transactions (24). These data include overdoses and hospitalizations, substance use disorder diagnoses, and prescriptions for medications like opioid agonist treatment (OAT) (24). Raw data are recorded under the responsibility of health information custodians, including physicians, hospitals, pharmacies, and government agencies, such as the Ontario Ministry of Health and Long-Term Care, and the Chief Coroner of Ontario. Under Ontario’s privacy laws (e.g., Personal Health Information Protection Act [PHIPA]), health information custodians are authorized to collect, use, and disclose personal health information for certain purposes, such as for use in public health management (24). In general, these data are recorded using diagnoses/variable codes from source code systems like the Diagnostic and Statistical Manual of Mental Disorders [DSM-5] Criteria and the International Statistical Classification of Diseases and Related Health Problems [ICD-9 or ICD-10] (24, 29). They are then inputted into national administrative health databases, such as the Discharge Abstract Database (DAD) and the National Ambulatory Care Reporting System (NACRS), along with provincial-specific databases like the Narcotics Monitoring System (NMS) and the OMHRS (24). See Supplementary Table 1 for a non-exhaustive list of national and provincial administrative health databases, outlining relevant unregulated substance use indicators such as emergency department visits, hospitalizations, treatment utilization and dispensed medications.

#### 3.1.1 Management and storage of administrative unregulated substance use health data in Ontario

In Ontario, administrative health databases are managed and stored by provincial data centers such as ICES, and independent organizations, such as CIHI. These organizations are designated as “prescribed entities” under PHIPA and are therefore authorized to collect individual-level personal health information from custodians without consent (30). Since individuals may access various health services across the healthcare system, their information may be recorded in multiple administrative health databases. Data are then anonymized by assigning facility-unique identifiers, such as those derived from Ontario’s universal health coverage plan (i.e., the Ontario Health Insurance Plan [OHIP]) (31, 32). This anonymization process ensures that individual records can be de-identified and linked across multiple databases, providing a more comprehensive view of an individual’s interactions with the healthcare system (5).

ICES and CIHI oversee numerous national and provincial administrative health databases, though the exact number of databases held at any given time varies as new databases are created and others are updated or consolidated. Together, they manage de-identified and linkable unregulated substance use-related health data (including diagnoses, harms, and treatment) for over 14 million people in Ontario covered under OHIP (24, 31, 32).

#### 3.1.2 Access to and dissemination of administrative unregulated substance use health data in Ontario

Accessing unregulated substance use-specific administrative health data from national and provincial administrative health databases involves navigating complex regulations. According to PHIPA, only prescribed entities like ICES and CIHI have legal authorization to access and disclose personal health information for purposes such as healthcare system planning, evaluation, and monitoring (30).

As custodians of administrative health data, prescribed entities must ensure the privacy and confidentiality of these data. They control access and use through data sharing agreements with the original data providers, and through policies and processes that have been approved by the Information and Privacy Commissioner of Ontario (32). External entities or researchers seeking access to these data for research purposes must submit formal data access requests to ICES or CIHI, providing detailed project plans that include research purposes, intended audiences, and relevant research ethics board approvals (32). These requests undergo rigorous and time-intensive reviews and evaluations based on methodology, feasibility, timeline, and ethical or privacy considerations (6).

Additionally, several online sources regularly access, analyze, and publish unregulated substance use-specific data for Ontario. Examples include ODPRN’s Opioid Indicator Tool, PHO’s Interactive Opioid Tool, the Public Health Agency of Canada’s Opioid-and Stimulant-Related Harms Interactive Tool, and the Canadian Substance Use Costs and Harms (CSUCH) Tool (1, 26, 27, 33). These sources present unregulated substance use-related health data on relevant indicators such as opioid-related ED visits, hospitalizations, OAT prescriptions, harm reduction services, substance use-related injuries, and deaths (1, 26, 27, 33). Moreover, some local and municipal health units also publish dashboards, such as Toronto Public Health’s Toronto Overdose Information System, which provides municipal-level health data on unregulated substance use in the city, including hospital visits and opioid toxicity deaths (34). See Supplementary Table 2 for a non-exhaustive list of select publishers of unregulated substance use-specific health data in Ontario, including a list of relevant data publishers, data sources, indicators, scope, and frequency of data updates.

### 3.2 Non-administrative unregulated substance use health data in Ontario

Governments, independent agencies, and organizations also gather non-administrative unregulated substance use-specific health data (28). These are often obtained through representative cross-sectional surveys, capturing self-reported substance use prevalence, problematic use, and qualitative insights into behaviors or social determinants of health at both the provincial and national levels (28). For instance, in Ontario, CAMH conducts surveys such as the Ontario Student Drug Use and Health Survey for youth and the CAMH Monitor for adults, exploring substance use prevalence, harms, behaviors, and attitudes (25, 35). See Supplementary Table 3 for a non-exhaustive list of select sources collecting non-administrative unregulated substance use health data in Ontario.

#### 3.2.1 Management, storage, and dissemination of non-administrative unregulated substance use health data in Ontario

Non-administrative unregulated substance use health data, typically obtained from surveys, are generally managed and stored by the organization conducting the survey. These entities hold legal authority to store and utilize the data they collect for research purposes, as well as to publicly share their analyses while ensuring compliance with privacy regulations (36). For example, Statistics Canada provides data access to external researchers upon request, often with an associated cost, subject to approval under the *Statistics Act,* a Canadian federal law that governs the collection, analysis, and dissemination of statistical information by the Government of Canada (37).

Organizations routinely distribute aggregated non-administrative health data on unregulated substance use after completing each data collection cycle. Researchers can then integrate data from multiple sources into their analyses (33).

Independent researchers, academic institutions, and organizations (e.g., harm reduction networks) also contribute to the collection and dissemination of health data on substance use. For example, the Canadian Research Initiative in Substance Matters (CRISM) collects and shares data on unregulated substance use prevalence, treatment and service utilization, access, and harms among people who use drugs (PWUD) both within the province and nationally (38, 39).

### 3.3 Administrative and non-administrative race-based health data in Ontario

Both administrative and non-administrative health data are also collected on racialized individuals and communities in Ontario (40). Much of these data are collected in the same ways (i.e., through healthcare visits and surveys), however, there are specific processes that need to be taken into consideration during their collection and dissemination. Specifically, unregulated substance use health data collected on First Nations, Inuit, and Métis peoples in Ontario must be collected, protected, used, and shared in accordance with the First Nations’ principles of ownership, control, access and possession (OCAP), which supports Indigenous rights to self-determination (40, 41). While Métis and Inuit communities also uphold data sovereignty, they may apply distinct governance principles developed through their own representative bodies. Similarly, data collected on Black individuals are governed by EGAP (Engagement Governance, Access and Protection) principles (42).

These frameworks ensure that Indigenous and Black communities have a right to any data that identifies their communities, and a right to determine how these data are used. As such, all data-collecting and data-holding organizations that collect, use, or manage these data have a responsibility to uphold data sovereignty (40, 41). For instance, ICES collaborates closely with First Nations, Inuit, and Métis partners to implement unique data sharing agreements, and Indigenous-led processes for the use and analyses of any data that identifies Indigenous people or communities (41). At ICES, any requests involving the use of Indigenous data, including in provincial-level analyses, are received and approved by a First Nations Data Governance Committee (41). This process is further governed through separate agreements with individual First Nations communities (e.g., Métis Nation of Ontario) and their respective communities or regions (41).

As such, initiatives to collect and disseminate unregulated substance use data on Indigenous or Black communities are generally led by these groups, and are tailored for their communities, upholding their rights to data ownership and self-determination (40). For instance, the Chiefs of Ontario administer a provincial survey assessing unregulated substance use prevalence, behaviors, health determinants, and service utilization among First Nations individuals living on reserves, as part of the First Nations Information Governance Center’s National Regional Health Survey (43).

## 4 Challenges and limitations associated with unregulated substance use-specific health data in Ontario

Ontario’s unregulated substance use health data landscape is complex, leading to challenges such as data fragmentation, redundancy, information gaps, and delays in collection and dissemination processes. These challenges hinder seamless data sharing and collaboration among stakeholders, thereby limiting the effectiveness of monitoring and intervention efforts in addressing unregulated substance use in the province. The following sections describe these challenges in detail and their impact on efforts to combat substance use-related harms.

### 4.1 Data fragmentation and redundancy

Fragmentation refers to the dispersion or division of data across multiple sources, while redundancy involves the duplication of data collection efforts or the reporting of similar data by different entities, both of which lead to inefficiencies (11, 44).

The decentralized and unsystematic nature of healthcare and social service delivery in Ontario results in piecemeal data collection and management, where information is scattered and fragmented across different sources and regions, and not integrated into cohesive datasets (11). Multiple organizations collect health data related to unregulated substance use, often using different measures, methodologies, and terminologies. This lack of standardization hinders comparability and interoperability, making it difficult to consistently report indicators and understand substance use issues across different regions, groups, or time periods (11, 16, 44, 45). For example, the wording and structure of survey questions can influence respondent interpretation, and subsequently, the answers obtained (28, 46). Moreover, there are notable differences in definitions, measures, and coding systems used between organizations and jurisdictions (7, 45, 47, 48). This heterogeneity limits the ability to compare data and service performance across organizations in Ontario, as well as between Ontario and other Canadian jurisdictions (7, 8). For instance, the definition and categorization of “unregulated overdose death” can vary across provinces, such as the origin of the associated drugs (i.e., whether it was unregulated or not), and the involvement of other regulated or unregulated substances that may have contributed to the death (1). In addition, provinces and territories may utilize different versions and levels of precision of diagnosis code systems in administrative health databases (7). For example, diagnoses on physician claims in Ontario are coded using ICD-8 codes, while Alberta and Manitoba use ICD-9 codes, with five digit- and three digit-levels of precision, respectively (7). These differences limit the ability to standardize or adapt diagnostic algorithms to conduct accurate national-level analyses (7).

Redundant data collection and reporting efforts also arise when organizations independently collect and/or disseminate the same or similar data without coordination (11). For example, PHO’s Interactive Opioid Tool and the Public Health Agency of Canada’s Opioid-and Stimulant-related Harms in Canada tool both report on opioid-related deaths and hospitalizations occurring in Ontario (1, 26). These data are not only redundant, but are also often marginally different between platforms, resulting in discrepancies and confusion. For instance, PHO’s Interactive Opioid Tool records the number of opioid overdose deaths that occurred in Ontario in 2022 as 2,535, whereas Health Canada reports the number as 2,531, likely due to differences in the data abstraction processes and timing (1, 26). Although the difference is diminutive, this discrepancy leads to uncertainty and limits the capacity to integrate multiple datasets to generate population estimates.

Integrating unregulated substance use health data from various data sources presents further challenges, particularly when linking administrative and non-administrative health data. Personal identifiers such as names, dates of birth, OHIP numbers, or postal codes are typically used to link information across different sources. While administrative data can often be linked deterministically using these identifiers, this method is limited for non-administrative or external data that may lack such identifiers (32, 49, 50). In these instances, probabilistic linkage is used; however, this method has its limitations (50–52). Using personal identifiers can lead to errors and instances of falsely-matched or unmatched records, as these identifiers are often transient (e.g., due to changes in residence), or may not be unique (e.g., if multiple individuals share the same name or date of birth) (51, 52). The inability to link complete samples can affect analyses, introduce selection bias, and reduce the generalizability of results, thereby impeding the quality of linked data (52).

### 4.2 Data collection gaps

In addition to fragmentation and redundancy, substantial gaps exist in the collection of unregulated substance use-specific health data in Ontario. These include the absence of comprehensive data on treatment uptake, drug use profiles and patterns, population prevalence estimates, data on specific subpopulations (e.g., those who are incarcerated or unhoused), influences of social determinants of health, and disaggregated data by socio-demographic characteristics such as gender, ethnicity, and socio-economic status. These deficiencies hinder the ability to fully understand and address the scope and nuances of unregulated substance use in the province.

#### 4.2.1 Gaps in data on substance use treatment

The lack of comprehensive data on substance use treatment in Ontario can be largely attributed to the fact that many substance use treatment facilities in the province are privately funded (16, 53, 54). Although some data on the availability and types of substance use treatment services are captured in administrative databases such as the Drug and Alcohol Treatment Information System (DATIS), these data are limited to publicly-funded treatment services and are therefore incomplete (55). Administrative health databases in Ontario do not capture diagnoses and services delivered outside of Ontario’s public healthcare insurance plans, as current provincial data privacy laws do not govern the collection, use, and disclosure of personal information by institutions and health providers outside the public sector (49, 50, 56). This includes any data collected from private health organizations or independent organizations, such as privately or donor-funded recovery treatments and support services, as well as community-level resources such as shelters and harm reduction services, leaving a significant information gap on the uptake of these treatments and services (56). Moreover, this gap is compounded by variability in definitions of treatment and treatment types (e.g., residential treatment, detoxification programs, outpatient programs), differences in surveillance indicators among programs, and inadequate efforts to disseminate these data where available (16, 54). For instance, current healthcare accreditation programs lack harm reduction and treatment performance measures, often limiting organizations’ knowledge of valuable surveillance indicators required to improve outcomes (10). Additionally, national efforts to collect and share publicly funded substance use treatment utilization data in Canada, including DATIS data from Ontario, have stalled. For example, the National Treatment Indicators project, has not been updated since its last published report in 2021, which covered data from 2016 to 2018 (57).

#### 4.2.2 Gaps in data on specific population subgroups

There are also information gaps in unregulated substance use surveillance and harm assessments for specific population subgroups. These include correctional populations, migrants/asylum seekers, people experiencing homelessness, veterans, and other vulnerable or hard-to-reach populations who are often excluded from national cross-sectional surveys due to sampling bias (e.g., telephone or permanent mailing address requirements) (28, 53, 58, 59). Specifically, data on substance use prevalence and estimates of substance use disorders among individuals who are incarcerated or unhoused are sparse, due to their underrepresentation in national surveys and administrative health datasets (49, 53, 59). Other data gaps among specific subpopulations may be related to limited resource and training capacity for the collection, management, and sharing of data among organizations who commonly work with these communities (16, 18, 60). For instance, organizations may lack the ability to collect comprehensive and inclusive data on their clients, resulting in the absence of data on these groups (16, 18, 60, 61).

#### 4.2.3 Gaps in data on social determinants of unregulated substance use and harms

In addition, addressing the causes and harms of unregulated substance use requires comprehensive information on the physical, mental, and social influences of health, including social determinants of health (2, 3, 62). These data are important for policymakers to gain a holistic understanding of the intersecting factors associated with unregulated substance use, and to effectively address the multifaceted needs of populations and specific subgroups (2, 3, 62). This typically involves collecting information from sectors, ministries, and organizations outside of the health sector, such as children and youth services, community and social services, correctional services, immigration, and housing (63). However, in Ontario, existing data collected across sectors and ministries are often overlooked in larger-scale provincial health data strategies, and are not consistently included in medical records or administrative health data (63). Current cross-sectoral data linkage efforts are typically ad-hoc and program-specific, missing opportunities for sustained integration, particularly with socio-demographic data (3, 50, 51, 53, 62, 63). The lack of an integrated approach to data collection and analysis across the public sector impedes the ability to draw important longitudinal connections and insights between health and social factors that influence substance use (63).

#### 4.2.4 Gaps in disaggregated data

Additional issues that result in data gaps include instances where available data are broadly aggregated (2, 18, 44, 64). Limitations on how data are collected, organized, and presented can impede efforts to identify emerging issues and target interventions effectively and precisely (2, 18, 44, 64). For instance, there is a distinct need for disaggregated data which would allow for analyses that are more sensitive and would fill critical gaps in understanding potential inequities in impacts of unregulated substance use among different subgroups (2, 18, 44, 64). Additionally, the collection of more granular data can help address issues related to absent data in linkages, particularly where records cannot be matched due to insufficient identifiers, thereby reducing bias and improving the accuracy of population estimates (65). Strengthening these data linkages is critical for identifying service gaps, targeting interventions, and ensuring that harm reduction, treatment, and prevention strategies are reaching populations most at risk of overdose or drug-related harms (65).

#### 4.2.5 Gaps in data on ecological drug use

There is also a notable lack of ecological drug use data in Ontario, including detailed information on the types of drugs in circulation over time and by location, as well as population-level prevalence estimates of people who use unregulated drugs. Reliable data on drug use profiles, such as substance type and source, mode of use (e.g., injection versus inhalation), duration, and frequency of use, remain limited. Currently, these data are primarily derived from population-based surveys, which rely on self-reports and are prone to several biases (66). Moreover, the rapidly evolving unregulated drug market introduces novel substances that are often not systematically captured in surveys (28). When they are captured, substances may be misclassified or classified under broad categories (e.g., fentanyl-related compounds), limiting disaggregation by drug type and mode of use (28). Additionally, inconsistent or outdated terminology (e.g., drug names) across surveys, which may not reflect street names used by PWUD, further compromises data accuracy and completeness (28).

#### 4.2.6 Gaps in data on socio-demographic and equity-related characteristics

In addition, existing unregulated substance use health data in Ontario often suffer from broad categorization of socio-demographic identifiers, such as racial or ethnic classifications. For instance, data on racial and ethnic origin are often grouped into non-specific categories such as ‘Asian’, ‘Indigenous’, or ‘Visible Minority’ which can obfuscate potential differences, disparities, and inequities within these populations (58, 67). The use of aggregate socio-demographic and race-based data is largely due to the limited collection of these data within the health system more broadly (68). Currently, Ontario mandates the collection of standardized race-based data through the *Anti-Racism Act* for certain public sector organizations (e.g., child and youth services, and correctional services), but not universally across services within the health sector (67, 69). Moreover, the collection and use of data on historically-racialized groups such as Indigenous or Black communities must occur within the context of the data sovereignty principles established by these communities (e.g., OCAP and EGAP, as described above), and ensure their equitable involvement in any data activities that involve their communities (40–42). As such, the meaningful engagement of these groups, or lack thereof, in the design of data initiatives can lead to varying capacities to collect and use data on these sub-populations (40–42, 61). This can lead to inconsistent identifiers and indicators that may not accurately reflect their realities, effectively undermining the representation of these groups in population-level data (40, 61).

### 4.3 Data lags

Ontario also faces barriers to accessing timely data on unregulated substance use. Delays in the dissemination of both survey and administrative health data are common, and stem from internal limitations, data-sharing agreements, and varying schedules for data capture, uploading, abstraction, and publication (6, 70). Depending on the data source, data dissemination can range from several weeks to over a year (6, 70). For instance, while hospitalization discharge data are collected in real-time, delays often occur before they reach data holders (70). Survey data and overdose mortality data, based on coroner reports and toxicology results, also face long delays due to time-intensive validation and quality assurance processes (71).

Even once they are finalized, these data are often not accessible for broader research use. External researchers must undergo lengthy approval processes, which can be prolonged by study complexity, data quality concerns, and limited organizational capacity to manage requests (6, 10). Additional delays arise when linking datasets from different sources, which often requires negotiating data agreements, and transferring, cleaning, and computing data - processes that can be time-consuming and resource-intensive (6, 52). These processes are further slowed by misaligned data collection timelines (52, 72). As a result, access to linked administrative health datasets in Ontario (and Canada more broadly) can vary widely, ranging from a month to several years (6, 14).

Finally, there are limited tools available to rapidly submit and disseminate information on unregulated substance use for surveillance. As a result, analyzed data are often published *ad hoc* in peer-reviewed academic journals and reports, processes that can also be lengthy, often taking upwards of a year or more. This delay means that by the time research findings are published, the data may already be outdated (6, 10). This is particularly problematic in the context of the rapidly-evolving overdose crisis and shifting drug markets, where near real-time data is needed to inform effective response strategies to prevent harms.

## 5 Discussion

In the context of the overdose crisis, the findings from this review have important implications for improving the quality of the health data ecosystem at both the provincial and national level, particularly in relation to addressing unregulated drug use and enhancing health equity. These goals are in alignment with recent commitments to modernize and standardize health data systems (including mental health and addictions in particular) toward improving access to and stewardship of quality, de-identified, and comparable public health information. This information is imperative for research, reporting, and service improvement (2, 44, 73).

Overall, a wealth of health data and information is available from various provincial and national sources, offering the potential to generate valuable insights into unregulated substance use and related harms, both in Ontario and across Canada. These data form a strong foundation for a national surveillance system. At the provincial level, validation studies and quality assessments have confirmed the use of both administrative and survey-based health data in Ontario for research and surveillance (53, 74–79). For example, a recent validation study of case-ascertainment algorithms demonstrated the ability to accurately identify people who inject drugs in administrative health data in Ontario with high sensitivity and specificity for population-level health research (76). However, there remain critical issues regarding data fragmentation, redundancy, gaps, and lags, underscoring opportunities to optimize resources to address these challenges and enhance the usefulness of these health data. In particular, many crucial data sources are either fragmented, or are not structured in ways that enable rollout at a national level (9). For example, data collected by community-based harm reduction programs, Indigenous-led organizations, or local service providers are often stored in disparate systems, use non-standardized formats, or lack the infrastructure and resources required for consistent reporting. While these data are critical for understanding substance use trends among vulnerable populations, their piecemeal and siloed nature makes it challenging to integrate them into broader provincial and national surveillance initiatives. Addressing these limitations is not only crucial for accurately assessing healthcare needs, and improving outcomes within the province, but also for contributing to broader national objectives around integrated substance use surveillance (9).

Efforts to strengthen Ontario’s unregulated substance use data system can serve as a model for other jurisdictions, and help catalyze the implementation of a Canadian Drug Observatory (9). As Health Canada continues to explore the feasibility of a national drug observatory model, lessons from provincial systems, such as Ontario’s, can inform the development of standardized frameworks, governance models, and data-sharing protocols. Moving forward, efforts should focus on improving the collection, integration, and comparability of high-quality data across jurisdictions. These efforts must also reduce fragmentation, and ensure that data stewardship practices respect data sovereignty and uphold cultural integrity, particularly when managing individual-level health data.

We also note important limitations in collecting accurate and comparable data on unregulated substance use. Real-time data collection remains a challenge, and key indicators, such as drug composition, frequency of use, or use of harm reduction products (e.g., sterile needles), are often difficult to capture through administrative health datasets. These indicators are typically gathered through social or epidemiologic surveys instead, which, as noted earlier, face issues such as slow reporting, accuracy limitations, and difficulties in capturing less common or newly emerging substances in a rapidly evolving drug market (28). Even when such substances are included, sampling or coverage biases may result in low prevalence estimates or suppressed data due to privacy constraints (28). Additional limitations stem from respondent-related factors, such as memory recall, or the interpretation of survey questions, which can compromise data accuracy (28, 77). Despite these challenges, validation studies in Ontario have shown that people who inject drugs (PWID) report substance use behaviors with reasonable accuracy, supporting the value of self-reported data for capturing socio-demographic details and reducing classification bias (67). Furthermore, although this paper focuses on gaps and challenges in health data systems, it is crucial to consider complementary non-health data, such as arrests, sentencing, and incarceration records, which offer important context for understanding the underlying causes and contributors to the overdose crisis (80).

Recognizing these limitations, this paper offers key recommendations to strengthen data infrastructure across jurisdictions and advance the development of a timely, accurate, and equitable national substance use surveillance system. While Ontario serves as the case study for this review, the data challenges and lessons described are applicable across Canadian jurisdictions. As such, improving Ontario’s data infrastructure and surveillance capacity can offer scalable models for national implementation. Moreover, our findings may also hold relevance for other international regions with similar barriers to substance use data collection and integration. Addressing these systemic challenges at the provincial level is a critical step toward building a national observatory and surveillance system that supports evidence-based policy, mitigates harms, and promotes equitable access to care for diverse populations.

### 5.1 Recommendation 1: Establish data standardization and integration across sectors and jurisdictions

#### 5.1.1 Introduce frameworks and enhance policy mechanisms to promote data standardization

Standardized data collection and reporting measures are essential for generating reliable, accurate, and high-quality information that accurately reflects the unregulated substance use landscape. These measures can enhance the efficiency of data processes and enable comparability across jurisdictions within a national surveillance system. Our paper identified inconsistencies in terminology and metrics used by different organizations and sectors within Ontario, as well as across other provinces, territories, and international contexts (8). To address this issue, a national data dictionary should be developed to define terms, specify categories, standardize variable names and diagnostic codes, and align with industry and international reporting standards. This would improve the accuracy of national-level substance use data and enhance comparability with other countries (2, 16).

This data dictionary can ensure consistency in how key terms and outcomes, such as “treatment,” “adverse event,” “length of stay,” “substance use” (e.g., unregulated drugs vs. prescription medication), or “polysubstance use” (e.g., specifying which types/combinations of substances used), are defined and measured (16, 28). It should harmonize code systems, data element names, and value sets, clarifying which codes and values represent variables in electronic health reporting systems (7, 45). For example, identifying which codes/substances are included under the definition of “substance use,” along with specifying the mode of use (e.g., injection, inhalation, etc.) will promote shared meaning of concepts across systems, and allow for better disaggregation of data. In addition, guidelines for designing surveys that examine unregulated substance use behaviors would help reduce variations in research design, as well as reduce survey-related errors that impact data accuracy (28). Including street-level terminology (e.g., common names and forms of drugs) in these guidelines can improve the consistency and detail of the information recorded (28). Regular reviews and updates of terminology and indicators should be conducted to stay aligned with the evolving overdose epidemic in Canada, including changes in co-occurring opioid and non-opioid substance use over time.

#### 5.1.2 Integrate social, structural, and multisectoral data for effective substance use surveillance

The overdose crisis is multidimensional, involving rapidly changing dynamics across a wide range of interacting individuals, agencies, markets, and other societal entities and stakeholders. As such, to build a more comprehensive system that can effectively monitor and address emerging drug trends and harms, it is essential to broaden the surveillance scope to include the complex social and structural determinants of unregulated substance use. This includes strategies to streamline the integration of data from diverse sources, including across sectors, non-publicly funded organizations, and non-administrative health data sources (e.g., surveys). Leveraging information already routinely collected by these systems would require minimal investments, and would improve the identification of marginalized or hard-to-reach populations who are often socially excluded and underrepresented in administrative health data (e.g., people experiencing incarceration or homelessness, asylum-seekers, or sex workers) (63). For Ontario specifically, modernizing privacy legislation - such as PHIPA for health data and the Freedom of Information and Protection of Privacy Act (FIPPA) for data from other sectors - to include formal policies or data sharing agreements can help enable comprehensive data integration (63). Additionally, customizing data governance and privacy laws to include private health organizations that are not currently regulated under PHIPA would enhance the breadth of data collected (56, 63). Furthermore, provincial policies mandating or incentivizing standardized data practices (e.g., mandatory fields, public reporting requirements) can also accelerate alignment across Ontario’s health and social sectors. These strategies should be applied across other provinces and territories to ensure consistent reporting and harmonization of robust and comparable data for national monitoring and evaluation efforts.

#### 5.1.3 Strengthen data surveillance and integration capacity and modernize data governance frameworks

Effective data integration requires interoperable systems, compatible data formats, and standardized privacy protocols to support seamless exchange, integration, and storage across sectors (60, 63). In Ontario, data entities such as ICES have the technical capacity to link diverse datasets across provincial agencies with high linkage rates and extended coverage periods (50, 53, 81, 82). However, ICES’ designation as a prescribed entity under PHIPA restricts its scope to health information and to Ontario, limiting its ability to automatically link with data from other sectors, jurisdictions, and independent organizations (6, 83, 84). This constraint hinders cross-sectoral data integration and delays access to high-quality de-identified data (6). While ICES is expanding its partnerships to improve linkage capacity, policy changes that broaden its mandate to include sectors such as corrections, immigration, education, and children and youth services would enhance integration efforts (83). Enabling broader linkage would allow for more efficient data dissemination and provide policymakers with real-time comprehensive insights to design more responsive interventions, particularly for marginalized groups. For instance, linking opioid prescribing data with overdose and drug seizure records could support earlier detection of unregulated drug market shifts. Similarly, integrating social services or justice system data with treatment data could help identify high-risk groups and improve outreach strategies (22). Investing in real-time data integration, and strengthening surveillance infrastructure and data systems at the provincial level can serve as a scalable model for national infrastructure investments, contributing to a more cohesive pan-Canadian surveillance system.

Investing in local data capacity and workforce development, with a focus on data linkage is also key (60). These processes require skilled personnel with expertise in data technologies, research methods, and privacy legislation. Expanding training, technical support, and resourcing for data professionals is essential for the development of a national surveillance system (6, 60). Ontario’s Digital Health Playbook is one such initiative, guiding Ontario Health Teams in adopting digital health tools and training staff in data entry, reporting, and system navigation (85). Building equitable data capacity across the province will not only improve data quality and foster more timely data collection, but also enhance the granularity and representativeness of data to strengthen national surveillance capacity (85).

### 5.2 Recommendation 2: improve availability and accuracy of health data on unregulated substance use

#### 5.2.1 Engage PWUD in the design of data collection and surveillance strategies

Accurate data on unregulated substance use are essential for effective monitoring and surveillance efforts. As such, indicators and data collection methods related to unregulated substance use must be meaningfully-designed with significant input from PWUD communities (61). Participatory collaboration with PWUD in planning and development would help ensure that questions and data elements are conceptualized and interpreted consistently, and that the information captured reflects their lived experiences (28, 61). For instance, to reduce errors in survey design and improve efforts to identify new or emerging substances that may pose a public health threat, PWUD can provide insights on the terminology used in their communities and assist in designing questions that reflect diverse experiences with less commonly known substances. This involvement can substantially improve the accuracy and relevance of ecological and prevalence data used to monitor trends in unregulated substance use.

#### 5.2.2 Build long-term partnerships with equity-deserving groups and communities to develop data practices rooted in health equity and data sovereignty

The adoption of standardized socio-demographic data collection in routine clinical and health data processes - and across organizations and services involved in unregulated substance use care, both in Ontario and nationwide - can improve the ability to capture racial, ethnic, and socio-economic classifications. This, in turn, enhances the disaggregation of health data, enabling better characterization and identification of diverse population groups in both provincial and federal surveillance efforts. Policymakers and stakeholders should collaborate with provincial and national data-collecting organizations, such as Statistics Canada, to incorporate key indicators such as housing status, immigration status, socio-economic and linguistic categories, as well as specific categories representing all race, ethnicity, and sex and gender identities (3, 18, 67). To support this, guidance and training should be provided to care providers to ensure the safe and appropriate collection of race-based and identity data (68, 86).

However, it is crucial that the collection of any data adheres to community data sovereignty and governance processes established under OCAP or EGAP (18). Efforts to meaningfully engage with historically marginalized or racialized groups, such as Indigenous communities, should be prioritized when collecting health data concerning unregulated substance use among these groups, who are often underrepresented in data despite being disproportionately impacted by unregulated substance use (18, 40, 87). Organizations or researchers interested in collecting or analyzing data on these groups should ensure their equitable involvement throughout data collection, analysis, and reporting processes (40, 41, 61, 67, 87). These efforts can accurately capture their realities and social determinants of health, ensuring that surveillance initiatives reflect culturally relevant indicators and approaches, and promote data ownership (40, 41, 61, 67, 87). For example, tailored surveys like the Indigenous Peoples Survey and the First Nations Regional Health Surveys, developed with significant input from Indigenous communities and administered by Indigenous interviewers, capture comprehensive health and substance use data among Indigenous populations both on and off-reserve (40, 43, 88). These initiatives are critical for informing evidence-based policies and targeted interventions to mitigate the impact of unregulated substance use across diverse communities, thereby advancing health equity.

#### 5.2.3 Empowering communities for inclusive data stewardship

To further address data gaps and minimize the underrepresentation of particular subgroups in data collection, efforts are required to build data collection and management capacity among individuals from these communities and organizations that actively work with these groups (18). Bolstering training on data stewardship, privacy laws, research methods, and data analysis, among these communities would allow individuals to develop health indicators that accurately reflect their cultural backgrounds, lived experiences, and social determinants (40). This approach could foster trust among historically marginalized groups and promote autonomy and oversight in how their data are collected and utilized (40, 61). In addition, enhancing data infrastructure and equipping community organizations, such as harm reduction and treatment services, with basic data collection systems, can support the routine collection of quality data and linkage to other data, such as administrative health data (18, 60, 61). Investments in long-term, multi-disciplinary, collaborative initiatives involving government agencies, research institutes, and local/community-level organizations and researchers can improve infrastructure and provide the skilled personnel required for complex data linkage processes (60, 89). Inclusive and participatory collaboration can enhance data collection among marginalized populations, address crucial information gaps, and ensure that comprehensive and high-quality health data can be routinely collected, managed, and accessed efficiently (40, 41, 60). This can facilitate the usability and accessibility of disaggregated data, enabling a comprehensive understanding of health outcomes and social determinants of health. Together, these partnerships can provide frameworks and best practices that inform national engagement and reconciliation strategies in substance use surveillance efforts.

### 5.3 Recommendation 3: enhance collaboration and innovation to improve timely collection, analysis, and dissemination of high-quality health data

#### 5.3.1 Streamline data reporting, access, and governance across jurisdictions

Addressing the lack of timely and comprehensive information to effectively inform policy and public health decisions requires a coordinated effort to enhance the capacity of stakeholders to collect, access, and report high-quality substance use-related health data. Policymakers should explore legislative or regulatory mechanisms to facilitate quicker and more comprehensive reporting of reliable data. For example, in Arizona, United States, first responders and healthcare facilities are required to report overdose-related information within five days, enabling near real-time updates to their public dashboard (71).

Efforts should also focus on streamlining data access and integration, clarifying governance processes, and ensuring standardized reporting across jurisdictions. For researchers in Ontario, this includes clearer, and more transparent data access procedures within key data-holding organizations such as ICES, CIHI, and Statistics Canada, to reduce approval delays and support more responsive surveillance (6). For regulatory stakeholders, enhanced training in privacy and security protocols could improve the efficiency and consistency of data reviews, easing administrative bottlenecks that impede urgent research and policy analysis. Additionally, role-specific training for frontline healthcare and data management staff on standardized data entry and system protocols is essential to minimizing data entry errors, improving data quality, and enabling faster integration and analysis across platforms (6, 67, 90).

The establishment of a national drug surveillance system, and the ability to generate near real-time insights, fundamentally hinges on sustained collaboration across sectors and jurisdictions, and strong partnerships among federal, provincial, and territorial stakeholders (11). This includes researchers, data custodians, policymakers, frontline service providers, and communities most affected by unregulated drug use. Such a system must be grounded in a realistic understanding of what types of policies or programs require more timely, granular data (60, 63). For instance, evaluating the long-term impact of OAT or wraparound care models necessitates longitudinal data linkage across health, social, and justice sectors. Achieving these goals will require interoperable systems and data-sharing protocols that facilitate seamless integration, while maintaining strong privacy protections, both provincially and nationally. As an example, during the COVID-19 pandemic, the Ontario Health Data Platform enabled rapid data-sharing and collaboration across ministries, researchers, and healthcare institutions. This model demonstrated that collaborative data access to enable timely research and analysis can be achieved at the provincial level without compromising privacy standards. Applying similar principles to the overdose crisis could facilitate timely insights into emerging drug trends and intervention outcomes, while also allowing researchers across regions to replicate and build upon analyses, improving standardization and generalizability across provinces (3).

#### 5.3.2 Invest in innovative tools and infrastructure for rapid and inclusive data collection

Investments into innovative data strategies and modern infrastructure are also essential to fill critical gaps in existing surveillance systems. While promising developments, such as wastewater analysis, have expanded the toolkit for monitoring drug use trends, traditional analysis and monitoring methods remain slow, resource-intensive, and insufficient to meet the urgency and complexity of the substance use crisis (71). New strategies are needed to enhance the speed, reach, and depth of data collection and analysis nationally. For example, emerging approaches such as applying machine-learning techniques to analyze large-scale social media data, and deploying internet-based surveys, offer scalable and complementary tools to accelerate data collection and analysis, reach underrepresented populations, and deepen understanding of trends in unregulated drug markets, drug use, and their broader social, emotional, and cultural contexts (66, 91).

To meet the informational needs required for effective public health response, investments must support new infrastructure and approaches that prioritize timely and representative data, improved linkages across systems, and robust privacy protections (71). However, striking the right balance between data availability, efficiency, and confidentiality has long been a barrier to timely and comprehensive surveillance, and will only grow more complex with advancing technologies and increasing volumes of data. As novel opportunities to improve data processes continue to emerge, sustained innovation and cross-sector collaboration among public health and technology experts, data scientists, researchers, and policy makers, will be critical. These partnerships are essential to explore, validate, and responsibly integrate and operationalize these innovations into broader data strategies.

By enabling faster data collection and reporting, strengthening research infrastructure, fostering collaborative efforts, and integrating and scaling innovative approaches into national data surveillance strategies, these efforts can help support more agile, informed, and effective public health interventions at all levels (6).

### 5.4 Summary of recommendations

Effectively addressing unregulated substance use in Ontario, and across Canada, requires an integrated, coordinated, and innovative approach to health data management. The recommendations outlined above are interconnected and must be implemented collectively to support the design and sustainability of a national-level surveillance system, such as the Canadian Drugs Observatory currently under development by Health Canada (9). A robust national drug data system depends on the ability to standardize and integrate data across jurisdictions and sectors. Ensuring consistency, interoperability, and accessibility of data across health, social, and community-based systems is foundational to producing timely and actionable insights.

Timely, detailed data on drug toxicity trends can support early public health alerts and guide the geographic deployment of harm reduction services such as drug checking, naloxone distribution, and supervised consumption sites. Enhanced surveillance of treatment uptake and retention can help allocate resources more efficiently ensuring services are expanded where demand is highest. More granular socio-demographic data on those accessing harm reduction and treatment services would also enable policymakers to identify and address equity gaps, for example by refining outreach strategies to better reach marginalized populations disproportionately affected by the crisis. While ecological data on drug use patterns (e.g., substance types, modes of use), are well suited for targeted studies, the consistent collection of core indicators (e.g., overdoses, service utilization, mortality) is essential for routine monitoring and program evaluation.

Equally critical is enhancing the availability, representativeness, and accuracy of these data, closing important knowledge gaps, and ensuring that policy and program decisions are grounded in the lived realities of substance use. In parallel, fostering innovation and collaboration at the local, provincial, and national levels to accelerate and scale data collection, analysis, and reporting, can help transform how Canada tracks and responds to emerging public health challenges. Together, these efforts lay the groundwork for a more agile, inclusive, and evidence-informed surveillance ecosystem, one that is capable of driving meaningful decision-making to address the evolving unregulated substance use crisis, both in Ontario and nation-wide.

## 6 Conclusion

Comprehensive health data are essential for surveilling the changing unregulated substance use landscape, as well as for improving the delivery and quality of evidence-based strategies to effectively curb the overdose crisis in Ontario and beyond. However, systemic issues related to the collection, management, and reporting of unregulated substance use health data in Ontario persist, including data fragmentation, gaps, and lags, undermining the province’s ability to effectively monitor substance use trends and design timely and appropriate strategies to address high-priority needs. These provincial challenges also impede broader national efforts to establish a comprehensive surveillance system, by limiting the consistency, interoperability, and representativeness of data contributed across jurisdictions. Greater efforts are needed to enhance cross-sectoral standardization and integration of relevant data, improve data accuracy and availability, and reduce barriers to data collection, access, and dissemination through collaboration and innovation. Together, these strategies can increase the capacity to provide timely, comprehensive, disaggregated, and high-quality data on unregulated substance use at both the provincial and national levels, and support the implementation of a coordinated, high-quality, and responsive national substance use data surveillance system.
