# Supplementary material for: Exploring unregulated substance use health data in Ontario, Canada: Identifying gaps, addressing challenges, and uncovering opportunities
Source: Front Public Health. 2025 Aug 27;13:1477539. doi: 10.3389/fpubh.2025.1477539 (PMC12422062; doi:10.3389/fpubh.2025.1477539)
Supplement: Supplementary file 1 [file Data_Sheet_1.docx]

# **Supplementary Material**

Table S1. Overview of select national and provincial administrative health databases and relevant unregulated substance use-specific administrative health data collected

Table S2. Select health data publishers and indicators for unregulated substance use-related harms and treatment in Ontario

Table S3. Overview of Select Non-Administrative Unregulated Substance Use Data in Ontario.

# Table S1. Overview of select national and provincial administrative health databases and relevant unregulated substance use-specific administrative health data collected.*

| **Administrative Health Database** | **Description and Scope** | **Substance Use-Specific Outcomes Captured** |
| --- | --- | --- |
| National Ambulatory Care Reporting System (NACRS) | Captures data for hospital-based and community-based ambulatory care from all provinces and territories in Canada | - Emergency Department visits due to substance use, including due to cocaine, amphetamines, cannabis, stimulants, sedatives, hallucinogens |
| Discharge Abstract Database (DAD) | Contains demographic, administrative, and clinical data for all hospital inpatient discharges and day surgery interventions from all provinces and territories in Canada | - Emergency Department visits and hospitalizations due to substance use, including due to cocaine, amphetamines, cannabis, stimulants, sedatives, hallucinogens |
| Ontario Mental Health Reporting System Metadata (OMHRS) | Reports information on all individuals receiving adult mental health services in Ontario, including information about mental and physical health, social supports and service use, as well as care planning, outcome measurement, quality improvement and case-mix funding applications. | - Substance use hospitalizations, including due to cocaine, amphetamines, cannabis, stimulants, sedatives, hallucinogens - Utilization of health and social treatments and support services for substance use disorders |
| Drug and Alcohol Information System (DATIS) | Information system monitoring the number and types of publicly-funded substance use treatment services in Ontario. | - Characteristics of clients accessing services for substance use treatment - Utilization of treatment services for substance use disorders (including type of treatment accessed) |
| Narcotics Monitoring System (NMS) | Collects pharmacy and medication dispensing information about all controlled drugs from all dispensaries in Ontario | - Medications dispensed for the treatment of substance use disorders (Opioid Agonist Treatment medications) - Dispensing of methadone, buprenorphine containing products and/or slow release oral morphine (SROM), regardless of the payment type (eg, privately paid, public drug insurance) |

# * This is a non-exhaustive list of selective administrative health databases containing data specific to unregulated substance use in Ontario.

# Table S2. Select health data publishers and indicators for unregulated substance use-related harms and treatment in Ontario.*

| **Data Publisher** | **Data Sources** | **Relevant Data Indicators and Analysis** | **Scope** | **Update Frequency** |
| --- | --- | --- | --- | --- |
| **Public Health Agency of Canada:**  Opioid- and Stimulant-related Harms in Canada | - - Office of the Chief Coroner of Ontario - - NACRS - - DAD - - OMHRS | - **Overdose Deaths (Opioid and Stimulant)** - **Hospitalizations (Opioid and Stimulant)** - **Number of Emergency Medical Services responses to opioid overdoses** | National | Monthly |
| **Canadian Centre on Substance Use and Addiction:**  Canadian Substance Use Cost and Harms | - - CADS - - CCHS - - DAD - - Drug Analysis Services (Health Canada) | - **Overall Costs of Substance Use** - **Health Costs of Substance Use** - **Lost Productivity Costs of Substance Use** - **Criminal Justice Costs of Substance Use** - **All Other Costs of Substance Use** | National | Updated as data becomes available |
| **Ontario Drug Policy Research Network (ODPRN):** Ontario Opioid Indicator Tool | - - NACRS - - DAD - - Ontario Naloxone Program - - Needle Syringe Program - - Ontario Harm Reduction Distribution Program. | - **Opioids Dispensed for Pain** - **Opioid Agonist Therapy** - **Opioid-Related Harm** - **Provision of Harm Reduction (Naloxone, needles, bowl pipes, etc)** - **Rate of Prescription Stimulant Use** - **% of Individuals with Recent Psychiatric Visit** | Provincial | Quarterly |
| **Public Health Ontario (PHO):** Interactive Opioid Tool | - - NACRS - - DAD - - Office of the Chief Coroner of Ontario | - **Opioid-Related ED Visits** - **Opioid-Related Hospitalizations** - **Opioid-Related Deaths** - **Type of Drug Present at Death** - **Mortality from any benzodiazepine** - **Mortality from any stimulant** | Provincial | Quarterly |
| **ICES:**  Ontario Mental Health Systems Reporting Dashboard | - NACRS - DAD - OMHRS - OHIP - Postal Code Conversion - Registered Persons Database | - **Mental Health and Addictions-Related ED Visits** - **ED Visits for intentional self-injury** - **Mental Health and Addictions-Related Hospitalizations** - **Mental health and addictions-related outpatient visits** - **Mental health and addictions-related outpatient visits within 7 days after discharge** - **Mental health and addictions-related visits to ED as first point of contact** - **30-day mental health and addictions-related hospital readmissions** - **30-day mental health and addictions-related ED re-visits** - **Individuals seen by primary care provider, psychiatrist or paediatrician for mental health and addictions-related care** | Provincial | Monthly |
| **City of Toronto:**  Toronto Overdose Information System | - Toronto Paramedic Service - Toronto Public Health - Acute Care Enhanced Surveillance System - NACRS - DAD - The Works Supervised Consumption Service - PHO - Office of the Chief Coroner of Ontario - Ontario Harm Reduction Database | - **Suspected opioid overdose calls attended by Toronto Paramedic Services** - **Substance-related and suspected overdoses in Toronto Hospitals** - **ED visits due to opioid poisoning in Toronto hospitals** - **Hospitalizations due to opioid poisoning in Toronto hospitals** - **Deaths from opioid toxicity in Toronto** - **Visits to the Works Supervised Consumption Services in Toronto** | Regional (Toronto Public Health) | Updated as data becomes available |
| **Centre on Drug Policy Evaluation (CDPE):**  Toronto Disparities, Overdose and Treatment (T-DOT) | - Self-collected data - Registered Persons Database - NMS - DAD | - **Self-reported Opioid Agonist Treatment among PWUD** - **Uptake of harm reduction services among PWUD** - **Impact of harm reduction services on healthcare us** - **Impact of harm reduction services on health outcomes** | Regional  (Toronto) | Published as data becomes available |

# *This is a non-exhaustive list of select publishers of data specific to unregulated substance use in Ontario.

# Table S3. Overview of Select Non-Administrative Unregulated Substance Use Data in Ontario.*

| **Survey** | **Administering Organization** | **Description and Scope** | **Relevant Substance Use-Specific Indicators** | **Survey Cycle Frequency** |
| --- | --- | --- | --- | --- |
| Canadian Community Health Survey (CCHS) | Government of Canada | National cross-sectional survey that collects information related to health status, healthcare utilization and health determinants for Canadians age 18+ (excluding persons living on reserves and other Aboriginal settlements; full-time members of the Canadian Forces; correctional populations; and persons living in some Quebec health regions | - **Chronic Conditions and Mental Health Indicators (including Perceived Mental Health, and Perceived need for Mental Healthcare, and Pain or Discomfort)** - **Healthcare Required but not Received** - **Access to Health Services** - **Wait Time for Specialized Medical Services** - **Other risk factors for substance use** | Annual (Results published biennially) |
| Canadian Alcohol and Drugs Survey (CADS) | Government of Canada | National voluntary survey of all persons age 15+ living in Canada (except Yukon, NWT, Nunavut, and residents living on reserves, and correctional populations). Selected Households receive invitation letter. | - **Age of initiation of substance use** - **Frequency of use of illegal drugs, psychoactive pharmaceutical drugs** - **Potential harmful effects of illegal drugs, psychoactive pharmaceutical drugs** - **Received treatment for substance use** | Biennial |
| Canadian Student Tobacco, Alcohol and Drugs Survey (CSTADS) | Government of Canada | National voluntary survey of tobacco, alcohol, cannabis and drug use among Canadian students in grades 7 to 12 | - **Age of initiation of substance use** - **Frequency of use of illegal drugs, psychoactive pharmaceutical drugs** - **Prevalence of polysubstance use** - **Potential harmful effects of illegal drugs, psychoactive pharmaceutical drugs** - **Self-Reported Mental Health** - **Received treatment for substance use** | Biennial |
| Canadian Postsecondary Education Alcohol and Drug Use Survey (CPADS) | Government of Canada | National survey among recruited postsecondary students (aged 17-25) studying online or in person at a Canadian university, college, or CEGEP and living in Canada. | - **Frequency of use of Illegal Drugs** - **Behaviours and Harms associated with use of drugs** - **Reasons why substances were used** | Annual |
| Navigation of Mental Health and Substance Use Services Indicator | CIHI | National survey conducted amongst Canadians aged 15+ who accessed mental health and substance use services in the past year | - **Proportion of individuals (15+) who said they always or usually had the support necessary to move within and between mental health and substance use services in the past year once they accessed services** (Support refers to the receipt of guidance, assistance or resources from a professional, related to navigating mental health and substance use services.) | Annual |
| Early Intervention for Mental Health and Substance Use Among Children and Youth Indicator | CIHI | National survey conducted among individuals in Canada aged 13-24 who reported accessing at least one of the following services in the last 6 months | - **Proportion of individuals age 13 to 24 with early mental health and substance use needs who accessed community-based mental health and substance use services in the last 6 months** | Annual |
| Indigenous Peoples Survey | Government of Canada | National survey conducted among people aged 15+ living off reserve, Métis, and Inuit in Canada. | - **Frequency of use of street drugs, pain relievers** - **Mental health indicators (psychological distress, self-rated mental health, self harm)** - **Access to health services for Mental health** | Every 5 years |
| Canadian Veteran Health Survey | Government of Canada | National survey conducted Canadian veterans aged 18+ who were released the year prior to the survey collection cycle, and not re-enlisted or active. Those who are living in institutions or living on a reserve are excluded. | - **Frequency of use of street drugs, pain relievers** - **Mental health indicators (psychological distress, self-rated mental health, self harm)** | Every 2 years |
| Ontario Student Drug Use and Health Survey (OSDUHS) | CAMH | Provincial population survey conducted amongst students in grades 7 through 12 in over 200 elementary and secondary schools across Ontario | - **Frequency of use of opioids, other drugs** - **Mental health indicators (psychological distress, self-rated mental health, self harm)** - **Access to health services for Mental health** - **Unmet Need for Mental Health Supports** - **Reasons why substances were used** | Biennial |
| Centre for Addiction and Mental Health (CAMH) Monitor | CAMH | Ongoing provincial substance use and mental health survey of Ontario adults (aged 18 years +) | - **Frequency of use of opioids, other drugs** - **Behaviours and Harms associated with use of opioids, other drugs** - **Mental health indicators (psychological distress, prescribed medications for mental health, self-reported mental health)** | Biennial |
| [Ontario First Nations Regional Health Survey](https://chiefs-of-ontario.org/wp-content/uploads/2020/10/COO_RHS-PHASE-III-PEOPLES-REPORT.pdf) | Chiefs of Ontario | Provincial survey conducted among individuals living on-reserve in 26 First Nations communities across Ontario from ages 0-55+ (parent or guardian completed the survey for children) | - **Frequency of use of opioids, other drugs** - **Mental health indicators (psychological distress, self-rated mental health, self harm)** - **Access to health and support services for mental health and substance use** - **Reasons why substances were used** - **Socio-Economic Determinants of Health** | Every 5 years |
| Canadian Wastewater Survey(38) | Government of Canada | National survey and lab analysis conducted among wastewater treatment plants in several Canadian municipalities and regions (including Toronto, Ontario) | - **24 hr wastewater composite samples measuring Amphetamine, Cocaine, Codeine, Fentanyl, MDMA, Methadone, Methamphetamine, Morphine, and Oxycodone metabolites** - **Weather or other events that could have an impact on results** | Bimonthly |

# *This is a non-exhaustive list of select non-administrative data specific to unregulated substance use in Ontario.
